# The mechanism of Weiqi decoction treating gastric cancer: a work based on network pharmacology and experimental verification

**DOI:** 10.1186/s41065-025-00434-3

**Published:** 2025-04-21

**Authors:** Xu Huang, Zhihong Pan, Lei Shen, Huan Chen, Chang Chen, Tingting Lv, Yuzhou Mei

**Affiliations:** 1 Department of Gastroenterology, Renmin Hospital of Wuhan University, Wuhan, Hubei 430060 P.R. China; 2 Department of Gastroenterology, The First College of Clinical Medical Science, China Three Gorges University, Jiefang Road No. 2, Xiling District, Yichang, Hubei 443000 P.R. China

**Keywords:** Weiqi decoction, Gastric cancer, Network pharmacology, Pharmacological mechanisms

## Abstract

**Background:**

Weiqi Decoction (WQD) is an empirical prescription traditionally used in China for the treatment of precancerous gastric cancer (GC) lesions. This study aimed to elucidate the potential pharmacological mechanisms of WQD in GC therapy.

**Methods:**

Active ingredients, corresponding targets, and GC-related genes were identified using public databases. A protein–protein interaction (PPI) network was constructed via the STRING database, and functional enrichment analyses were conducted using the DAVID platform. Gene expression and survival analyses were performed using the GEPIA database. Molecular docking was conducted with AutoDock Vina and visualized using PyMOL. The effects of WQD on GC cell viability, proliferation, migration, and invasion were evaluated through CCK-8, colony formation, and Transwell assays.

**Results:**

WQD contained 43 active ingredients targeting 751 potential genes, including 458 GC-related targets. Quercetin, luteolin, and kaempferol were identified as key active compounds. PPI network analysis revealed nine core targets, including *TP53* and *SRC*, which may mediate the anti-GC effects of WQD. GO enrichment analysis indicated involvement in 726 biological processes, 91 cellular components, and 177 molecular functions, while KEGG pathway analysis suggested modulation of the AGE-RAGE, PI3K-Akt, and HIF-1 signaling pathways. GEPIA database analysis confirmed that *EP300*,* HSP90AA1*,* HSP90AB1*,* SRC*, and *TP53* were highly expressed in GC. Molecular docking demonstrated strong binding affinities between the key active compounds and core targets. In vitro experiments further validated that WQD extract inhibited GC cell viability, proliferation, migration, and invasion.

**Conclusion:**

WQD exhibits therapeutic potential against GC by regulating multiple targets and signaling pathways. These findings provide mechanistic insights into the pharmacological actions of WQD in GC treatment.

**Supplementary Information:**

The online version contains supplementary material available at 10.1186/s41065-025-00434-3.

## Introduction

Gastric cancer (GC) remains a major cause of cancer-related mortality. According to the GLOBOCAN project by the International Agency for Research on Cancer (IARC), over one million new cases are diagnosed globally each year [1, 2]. Current treatment strategies, including surgical resection, radiotherapy, chemotherapy, immunotherapy, and targeted therapies, have improved outcomes to some extent. However, the overall prognosis remains poor [3]. Therefore, developing effective anticancer agents with minimal side effects is of great clinical importance.

Traditional Chinese medicine (TCM) has been practiced in China for over 2,000 years and is increasingly recognized as a complementary therapy due to its efficacy, affordability, broad availability, and low toxicity [4]. Weiqi decoction (WQD), an empirical TCM formula, comprises seven medicinal herbs: Danggui (*Radix Angelicae Sinensis*), Huangqi (*Radix Astragali*), Dangshen (*Radix Codonopsis pilosulae*), Ezhu (*Rhizoma Curcumae*), Zhiqiao (*Fructus Aurantii*), Bayuezha (*Fructus Akebiae*), and Pugongying (*Taraxacum mongolicum*). Clinically, WQD has demonstrated therapeutic effects in treating atrophic gastritis [5, 6]. Recent studies suggest that WQD may also inhibit GC cell proliferation [6, 7], although the underlying mechanisms remain unclear.

Network pharmacology offers a powerful tool for elucidating the complex mechanisms of TCM [8–10]. Molecular docking further enables prediction and visualization of the structural characteristics, interaction patterns, and binding affinities between bioactive compounds and target proteins [11, 12]. This study utilizes network pharmacology and molecular docking to identify active compounds in WQD and their downstream targets and signaling pathways. Furthermore, in vitro models are employed to assess the effects of WQD extracts on GC cell malignancy.

## Materials and methods

### Identification of active ingredients and target genes of WQD

**Fig. 1:**
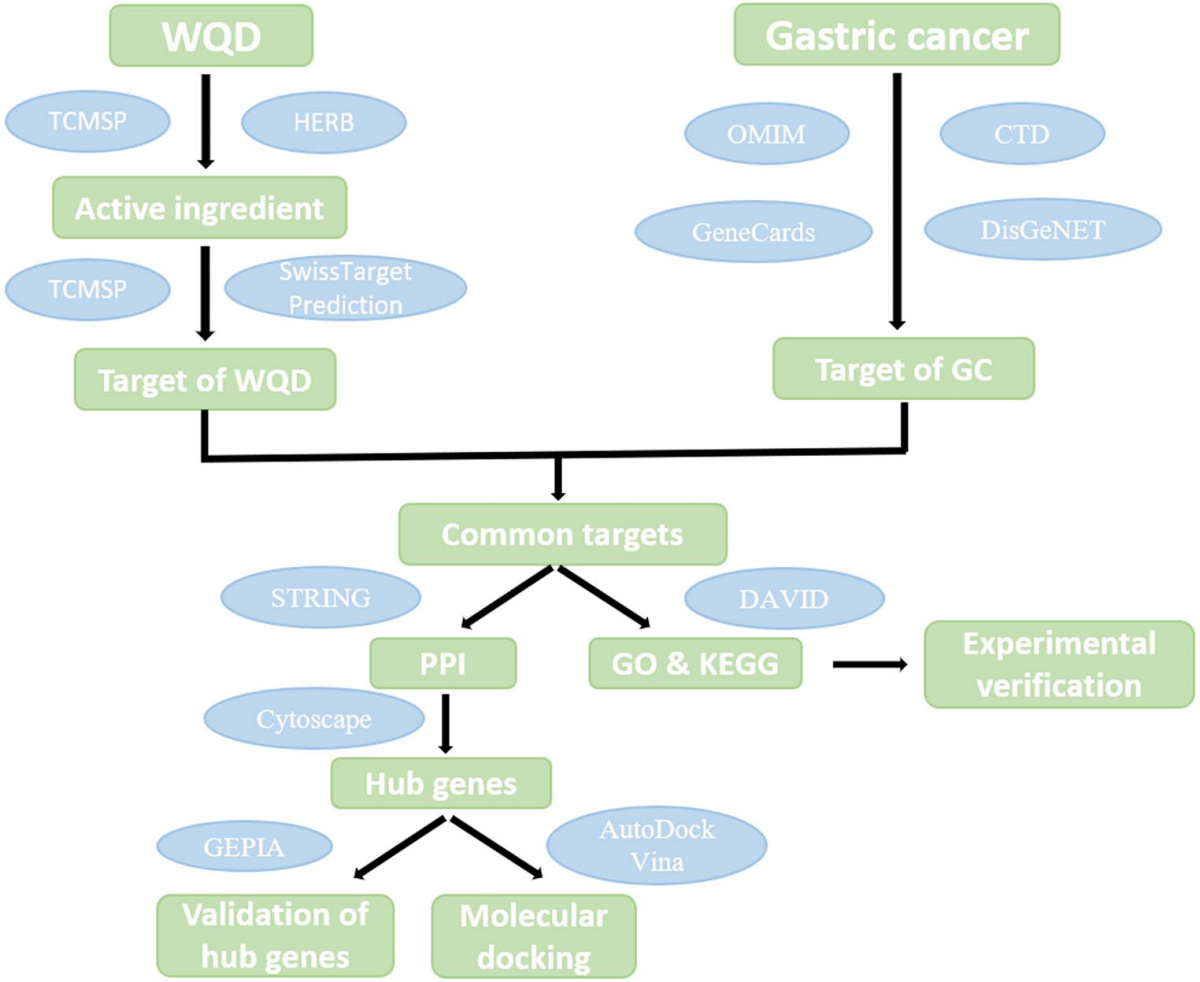
Workflow of the study

As illustrated in Fig. 1, WQD constituents were identified utilizing the Traditional Chinese Medicine Systems Pharmacology Database and Analysis Platform (TCMSP, http://tcmspw.com/tcmsp.php) and the HERB database (http://herb.ac.cn/) (Table 1). Active compounds were selected based on key pharmacokinetic properties, including oral bioavailability (OB) ≥ 30% and drug-likeness (DL) ≥ 0.18, which are crucial for absorption, distribution, metabolism, and excretion (ADME). The corresponding target genes of these compounds were predicted using both the TCMSP and SwissTargetPrediction databases (http://www.swisstargetprediction.ch/).

**Table 1:** The composition of WQD

Herb name	Latin name	Part used	Dosage (g)
Danggui	*Radix Angelicae Sinensis*	Root	12
Huangqi	*Radix Astragali*	Root	12
Dangshen	*Radix Codonopsis pilosulae*	Root	15
Ezhu	*Rhizoma Curcumae*	Root	15
Zhiqiao	*Fructus Aurantii*	Unripe fruit	15
Bayuezha	*Fructus Akebiae*	Dry and mature fruits	15
Pugongying	*Taraxacum mongolicum*	Aerial parts	30

### Acquisition of GC-associated genes

GC-related genes were collected utilizing “gastric cancer” as the keyword from the Comparative Toxicogenomics Database (CTD, https://ctdbase.org/, relevance score ≥ 27.82), DisGeNET (https://www.disgenet.org/, score ≥ 0.1), the Human Gene Database (GeneCards, https://www.genecards.org/, relevance score ≥ 10), and the Online Mendelian Inheritance in Man (OMIM) database (https://omim.org/).

### Construction of protein-protein interaction (PPI) network

The overlapping targets between WQD and GC were submitted to the Search Tool for the Retrieval of Interacting Genes/Proteins (STRING, https://string-db.org/), with the species set to Homo sapiens and a minimum confidence score threshold of > 0.90. The CytoNCA plug-in was employed to perform topological analysis. Core targets were identified by calculating betweenness centrality (BC), closeness centrality (CC), and degree centrality (DC), with the median values of each parameter used as selection thresholds.

### GO and KEGG pathway enrichment analyses

Functional enrichment of common targets was conducted utilizing the Database for Annotation, Visualization, and Integrated Discovery (DAVID, https://david.ncifcrf.gov/). Gene Ontology (GO) terms were classified into biological processes (BP), cellular components (CC), and molecular functions (MF), with a significance threshold set at *P* < 0.05 and false discovery rate (FDR) < 0.05. Kyoto Encyclopedia of Genes and Genomes (KEGG) pathway analysis was conducted with stricter criteria, using *P* < 0.01 and FDR < 0.01 to identify significantly enriched pathways.

### Expression characteristics and prognostic values of the core target genes

The mRNA expression levels and overall survival associations of core target genes were analyzed utilizing the GEPIA database (http://gepia2.cancer-pku.cn/#index).

### Molecular docking

Three-dimensional structures of the key target proteins were obtained from the RCSB PDB database (https://www.rcsb.org/). The 3D structures of active compounds in MOL2 or SDF format were retrieved from the TCMSP and PubChem databases. Protein and ligand structures were preprocessed using AutoDockTools-1.5.7, including dehydration and hydrogenation. Molecular docking was conducted with AutoDock Vina 1.5.7 to simulate ligand-target interactions. The docking results were visualized using PyMOL 2.4.0 and LigPlus.

### Preparation of WQD extract

The seven herbal components were prepared according to the prescription dosage (Table 1) and purchased from Shanghai Huayu Pharmaceutical Co., Ltd. The herbs were ground into powder and soaked in 95% ethanol for 12 h, followed by boiling for 2 h. The mixture was then filtered, and the liquid extract was collected. The residue underwent a second extraction by boiling in 95% ethanol for 2 h, followed by filtration. The combined extracts were concentrated and dried under a vacuum [13]. The resulting dried powder was dissolved in dimethyl sulfoxide and preserved at -20 °C.

### Cell culture

NCI-N87 and AGS gastric cancer cell lines were acquired from the American Type Culture Collection (ATCC, Rockville, MD, USA). Cells were cultured in RPMI-1640 medium enriched with 10% fetal bovine serum (Thermo Fisher, Shanghai, China) and 1% penicillin-streptomycin (Procell, Wuhan, China). The cells were maintained at 37 °C in a humidified incubator containing 5% CO₂.

### Cell counting kit 8 (CCK-8)

NCI-N87 and AGS cells were seeded into 96-well plates at a density of 5 × 10³ cells per well and treated with various concentrations of WQD extract (0, 200, 400, 600, 800, and 1000 µg/mL) for 48 h. Following treatment, 10 µL of CCK-8 solution (Dojindo, Tokyo, Japan) was added to each well and incubated for 2 h at 37 °C. Absorbance at 450 nm was measured using a Bio-Tek microplate reader (Winooski, VT, USA).

### Colony formation experiment

NCI-N87 and AGS cells were seeded in 6-well plates at a density of 1,000 cells per well. The culture medium was refreshed every three days. After 14 days, colonies were fixed with paraformaldehyde, stained with crystal violet (Sigma, St. Louis, MO, USA), rinsed with PBS, air-dried, and quantified using ImageJ software.

### Transwell experiment

The migration and invasion abilities of NCI-N87 and AGS cells were assessed using Transwell chambers (Corning, Madison, NY, USA), with or without Matrigel coating, respectively. Cells were resuspended in serum-free RPMI-1640 medium and seeded into the upper chambers. The lower chambers were filled with RPMI-1640 medium containing 15% fetal bovine serum as a chemoattractant. After 24 h of incubation, non-migratory or non-invasive cells on the upper surface were gently removed with a cotton swab. The remaining cells on the lower surface were fixed with paraformaldehyde, stained with crystal violet (Sigma), air-dried, and counted under a Nikon microscope (Tokyo, Japan).

### Quantitative real-time polymerase chain reaction (qRT-PCR)

Total RNA was extracted utilizing TRIzol reagent (Thermo Fisher, Shanghai, China) and reverse-transcribed into complementary DNA (cDNA) with the PrimeScript RT Master Mix (Takara, Dalian, China). qRT-PCR was conducted utilizing the SYBR Premix Ex Taq Kit (Takara) on an Applied Biosystems™ 7500 Fast Real-Time PCR System (Thermo Fisher). Gene expression levels were standardized to β-actin and determined via the 2^−ΔΔCt^ method [14]. The primers used in this study are listed in Supplementary Table S1.

### Statistical analysis

Data were reported as mean ± standard deviation (SD). Statistical analysis and graphing were performed utilizing GraphPad Prism 7.0 software. One-way analysis of variance (ANOVA) was conducted for comparisons among three or more groups. A P value of < 0.05 was considered statistically significant.

## Results

### Prediction of WQD targets

A total of 125 components from *Radix Angelicae Sinensis*, 87 from Radix *Astragali*, 134 from *Radix Codonopsis pilosulae*, 81 from *Rhizoma Curcumae* and 15 from *Fructus Aurantii* were retrieved from the TCMSP database. Additionally, 72 components from *Taraxacum mongolicum* and 7 from *Fructus Akebiae* were identified via the HERB database. Following screening based on oral bioavailability (OB) ≥ 30% and drug-likeness (DL) ≥ 0.18, 2 active compounds were retained from *Radix Astragali*, 18 from *Radix Angelicae Sinensis*, 18 from *Radix Codonopsis Pilosulae*, 3 from *Rhizoma Curcumae*, 5 from *Fructus Aurantii*, 5 from *Taraxacum Mongolicum*. No compounds meeting the criteria were identified from *Fructus Akebiae.* Several compounds were shared among different herbs. Beta-sitosterol (MOL000358) was identified in *Radix Angelicae Sinensis*, *Fructus Aurantii*, and *Taraxacum Mongolicum*. Stigmasterol (MOL000449) was present in *Radix Angelicae Sinensis* and *Radix Codonopsis Pilosulae*. Hederagenin (MOL000296) was detected in *Radix Astragali* and *Rhizoma Curcumae*, while Quercetin (MOL000098) was common in *Radix Astragali* and *Taraxacum Mongolicum*. Taraxerol (MOL006554) was identified in *Radix Codonopsis Pilosulae* and *Taraxacum Mongolicum* (Supplementary Table S1). After removing duplicates, 45 unique active components were obtained. Target prediction was performed using both the TCMSP and SwissTargetPrediction databases. Predicted target genes were successfully identified for 43 of the 45 compounds. However, no target genes were predicted for *chrysanthemaxanthin* (MOL004492) and *flavoxanthin* (MOL002680). In total, 751 genes were identified as potential targets of the active ingredients in WQD.

### Acquisition of potential targets for WQD in GC treatment

**Fig. 2:**
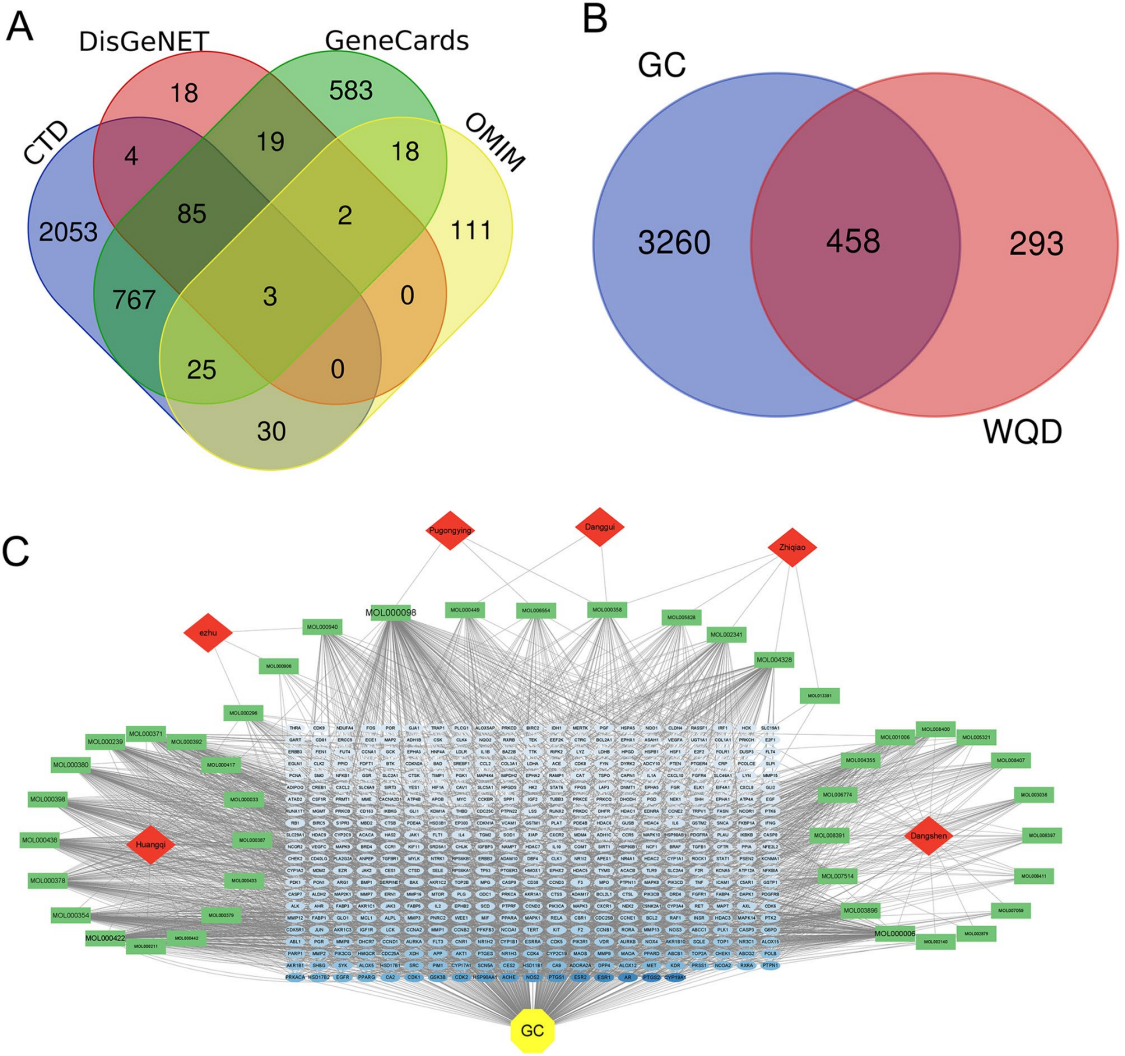
Identification of potential therapeutic targets of WQD in GC treatment. (**A**) The Venn diagram shows GC-related targets in CTD, DisGeNET, GeneCards, and OMIM databases. (**B**) The Venn diagram shows the intersection between drug targets and GC-related genes. (**C**) Drug - component - target - disease network. Node size represents the degree value. Green nodes represent bioactive components; red nodes represent drugs; blue nodes represent targets; and yellow nodes represent disease

A total of 2,967, 131, 1,502, and 189 GC-related genes were obtained from the CTD, DisGeNET, GeneCards, and OMIM databases, respectively, using “gastric cancer” as the keyword. After removing duplicates, 3,718 GC-related genes were obtained (Fig. 2A). The intersection between drug targets and GC-related genes resulted in 458 common targets (Fig. 2B). Cytoscape software was applied to visualize the drug-component-target-disease network, where node size corresponded to degree value (Fig. 2C). The resulting network comprised 509 nodes and 2,579 edges, including 6 herbs (drugs), 43 active compounds, 458 target genes, and one disease. Active components with high degree values were identified as key compounds, including quercetin (MOL000098, DC = 201), luteolin (MOL000006, DC = 130), and kaempferol (MOL000422, DC = 118).

### PPI network construction and hub genes screening

**Fig. 3:**
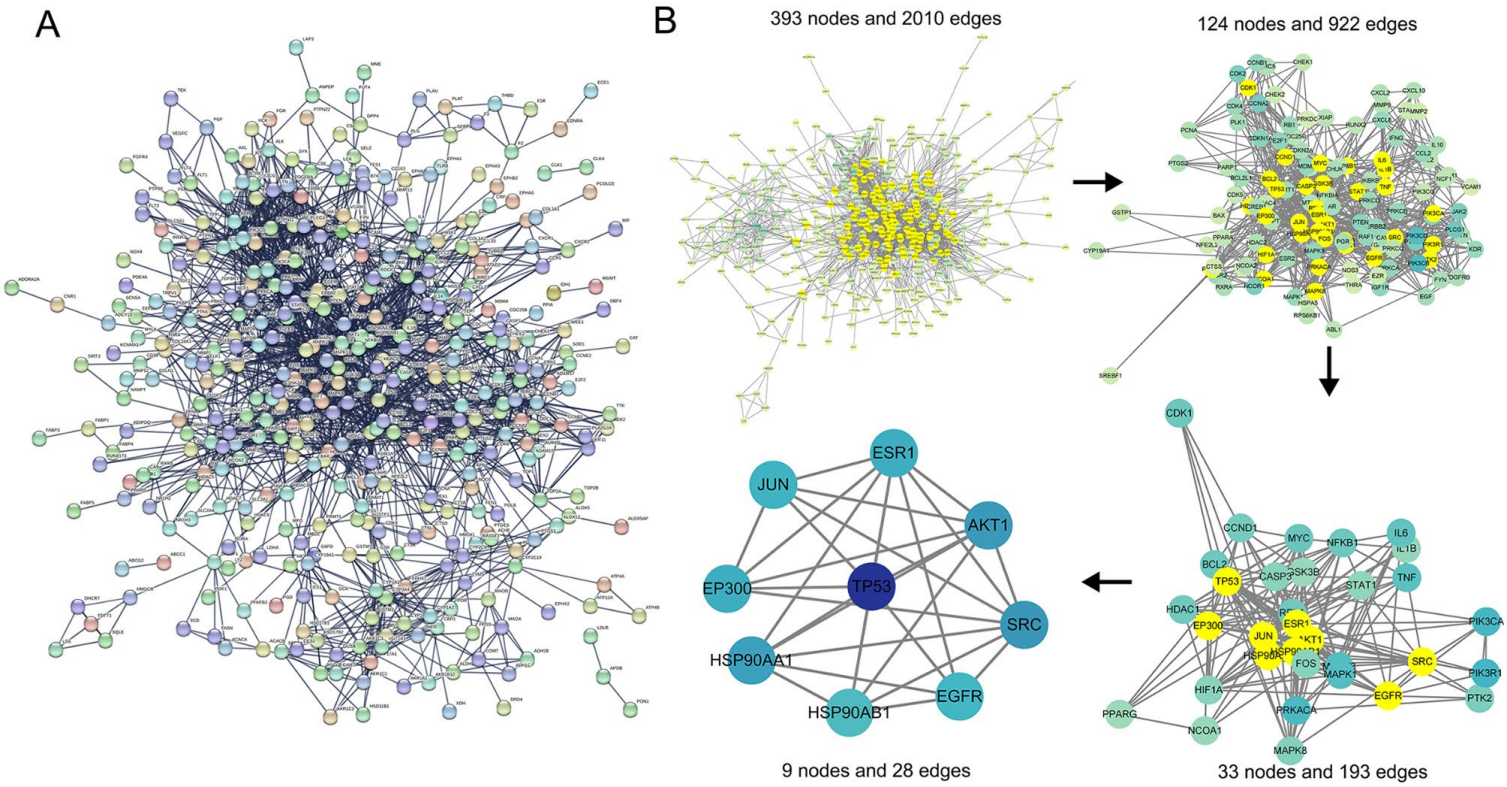
Construction of PPI network and screening of hub genes. (**A**) PPI network of 452 common targets generated using the STRING database. Each node represents a protein; the thickness of the edges reflects the confidence of the interaction. (**B**) Visualization and topological analysis of the PPI network using Cytoscape and CytoNCA plug-in. Darker node colors indicate higher degree values; yellow highlights the screened hub targets

A PPI network was established utilizing STRING based on the 458 common targets, resulting in 457 nodes and 2,015 edges (Fig. 3A). Topological analysis was performed utilizing the CytoNCA plug-in in Cytoscape software to calculate the values of BC, CC and DC. In the first round of screening, 124 genes were selected based on median values of BC, CC, and DC (Fig. 3B). A second screening step refined the selection to 33 targets (BC = 1,093.45, CC = 0.34, DC = 18.50). A final round of screening identified 9 core targets (BC = 3525.64, CC = 0.38, DC = 31.00), as shown in Table 2.

**Table 2:** The information of hub genes

Hub gene name	UniProt ID	Protein name	Degree	Closeness	Betweenness
TP53	P04637	Cellular tumor antigen p53	80	31206.36	0.44
SRC	P12931	Tyrosine-protein kinase SRC	50	8862.18	0.39
AKT1	P31749	Serine/threonine-protein kinase AKT	49	7160.32	0.41
HSP90AA1	P07900	heat shock protein 90 alpha family class A member 1	47	7772.064	0.40
ESR1	P03372	Estrogen receptor alpha	43	11670.73	0.42
EP300	Q09472	Histone acetyltransferase p300	43	12528.73	0.40
JUN	P05412	Transcription factor Jun	41	10720.17	0.42
EGFR	P00533	Epidermal growth factor receptor erbB1	40	5478.36	0.39
HSP90AB1	P08238	Heat shock protein HSP 90-beta	37	3660.27	0.39

### Results of GO and KEGG analyses

**Fig. 4:**
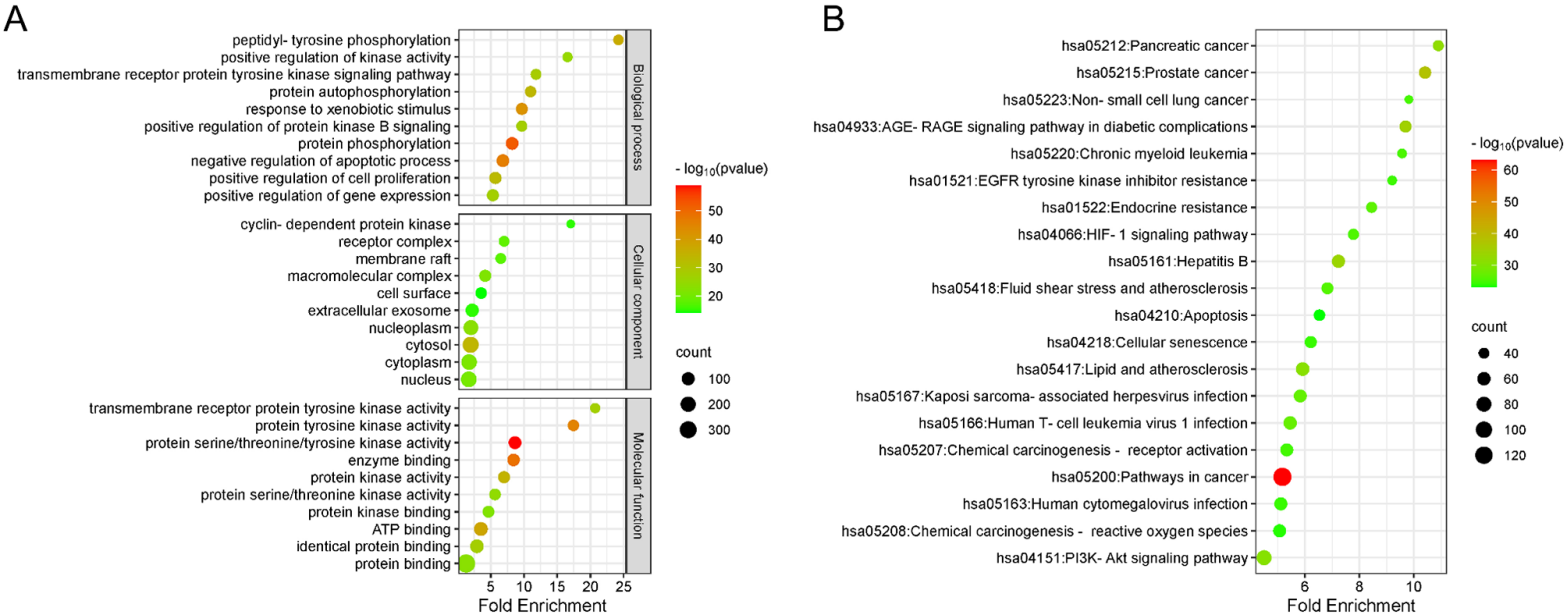
GO and KEGG enrichment analyses. (**A**) GO analysis of 452 common target genes. Circle size represents the number of genes; color intensity reflects the *p*-value. (**B**) KEGG enrichment analysis of 452 common target genes. Circle size and color indicate gene count and *p*-value, respectively

**Fig. 5:**
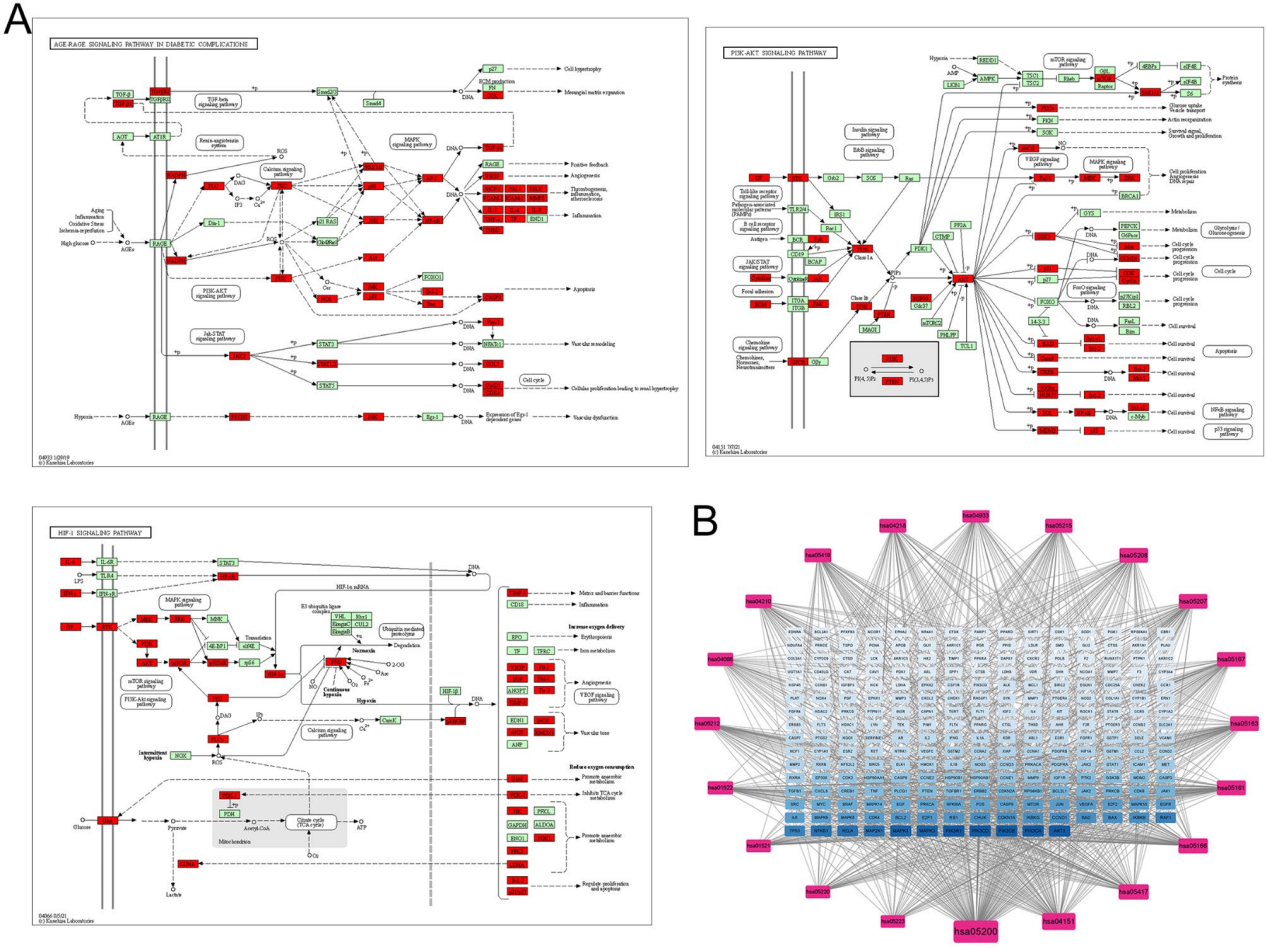
Gene pathway network. (**A**) AGE-RAGE signaling pathway in diabetic complications, PI3K-Akt signaling pathway, and HIF-1 signaling pathway. Red nodes indicate common targets; green nodes indicate non-overlapping targets. (**B**) Network diagram of common targets and 20 signaling pathways. Blue rectangles represent target genes; pink rectangles represent signaling pathways. Node size reflects the degree value

GO and KEGG pathway enrichment analysis were conducted for the 458 common target genes using the DAVID database. A total of 726 BP, 91 CC, and 177 MF were enriched (*P* < 0.05 and FDR < 0.05) (Fig. 4A). The most enriched BP terms included protein phosphorylation (GO:0006468), negative regulation of apoptosis (GO:0043066), and response to xenobiotic stimulus (GO:0009410). Enriched CC terms primarily included cytosol (GO:0005829), nucleoplasm (GO:0005654), and macromolecular complex (GO:0032991). The top MF terms were protein serine/threonine/tyrosine kinase activity (GO:0004712), enzyme binding (GO:0019899), and protein tyrosine kinase activity (GO:0004713). KEGG enrichment analysis revealed 181 significantly enriched pathways (*P* < 0.05, FDR < 0.05), among which key pathways included AGE-RAGE signaling in diabetic complications (hsa04933), PI3K-Akt signaling pathway (hsa04151), HIF-1 signaling pathway (hsa04066), and several cancer-related pathways (Figs. 4B and 5A). Topological analysis was conducted on the 236 genes involved in 20 pathways and the 236 target genes associated with WQD in GC treatment (Fig. 5B). Among the identified pathways, cancer-related pathways had the highest degree value (degree = 136), followed by the PI3K-Akt signaling pathway (degree = 80), suggesting that PI3K-Akt signaling plays a central role in WQD’s therapeutic effects against GC. These findings indicate that WQD exerts anti-GC effects by regulating multiple biological processes and signaling pathways.

### Clinical significance of core targets

**Fig. 6:**
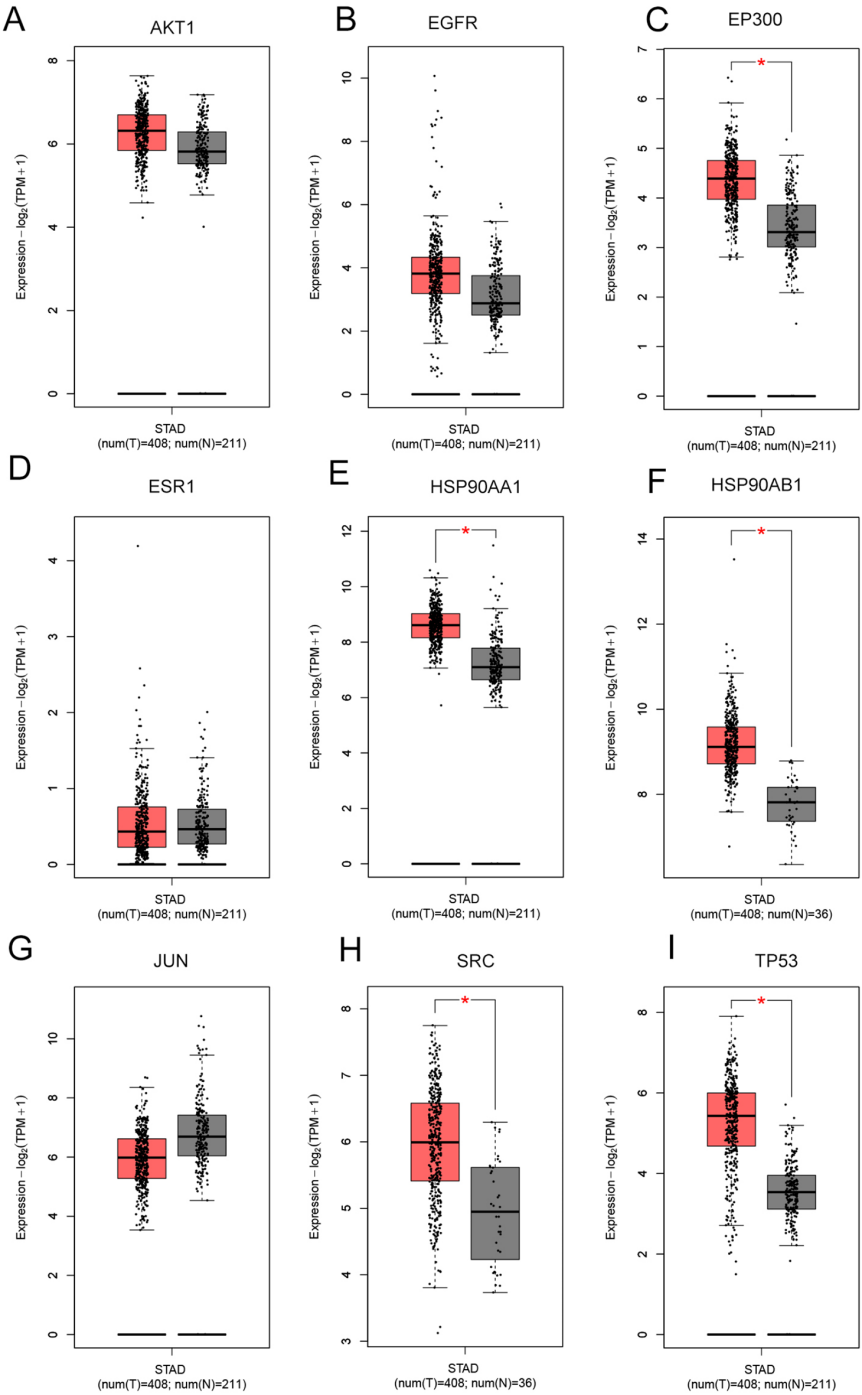
Expression levels of core targets in the GEPIA database in GC. Box plots show expression levels of AKT1 (**A**), EGFR (**B**), EP300 (**C**), ESR1 (**D**), HSP90AA1 (**E**), HSP90AB1 (**F**), JUN (**G**), SRC (**H**), and TP53 (**I**) in gastric cancer and adjacent normal tissues. * *P* < 0.05

**Fig. 7:**
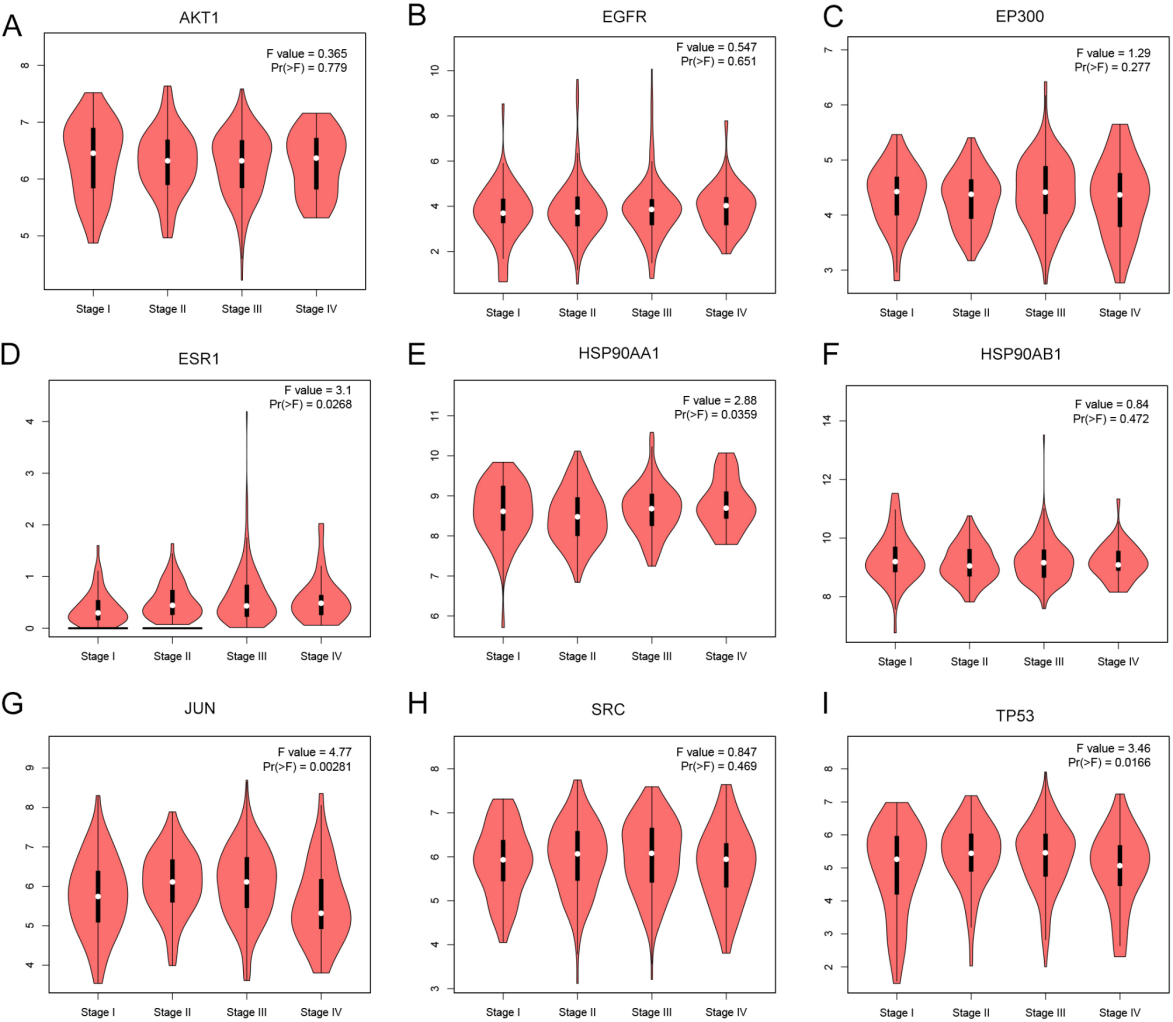
Expression levels of core targets in the GEPIA database at different stages. The violin diagrams show the expression levels of AKT1 (**A**), EGFR (**B**), EP300 (**C**), ESR1 (**D**), HSP90AA1 (**E**), HSP90AB1 (**F**), JUN (**G**), SRC (**H**) and TP53 (**I**) in different pathological stages of GC

**Fig. 8:**
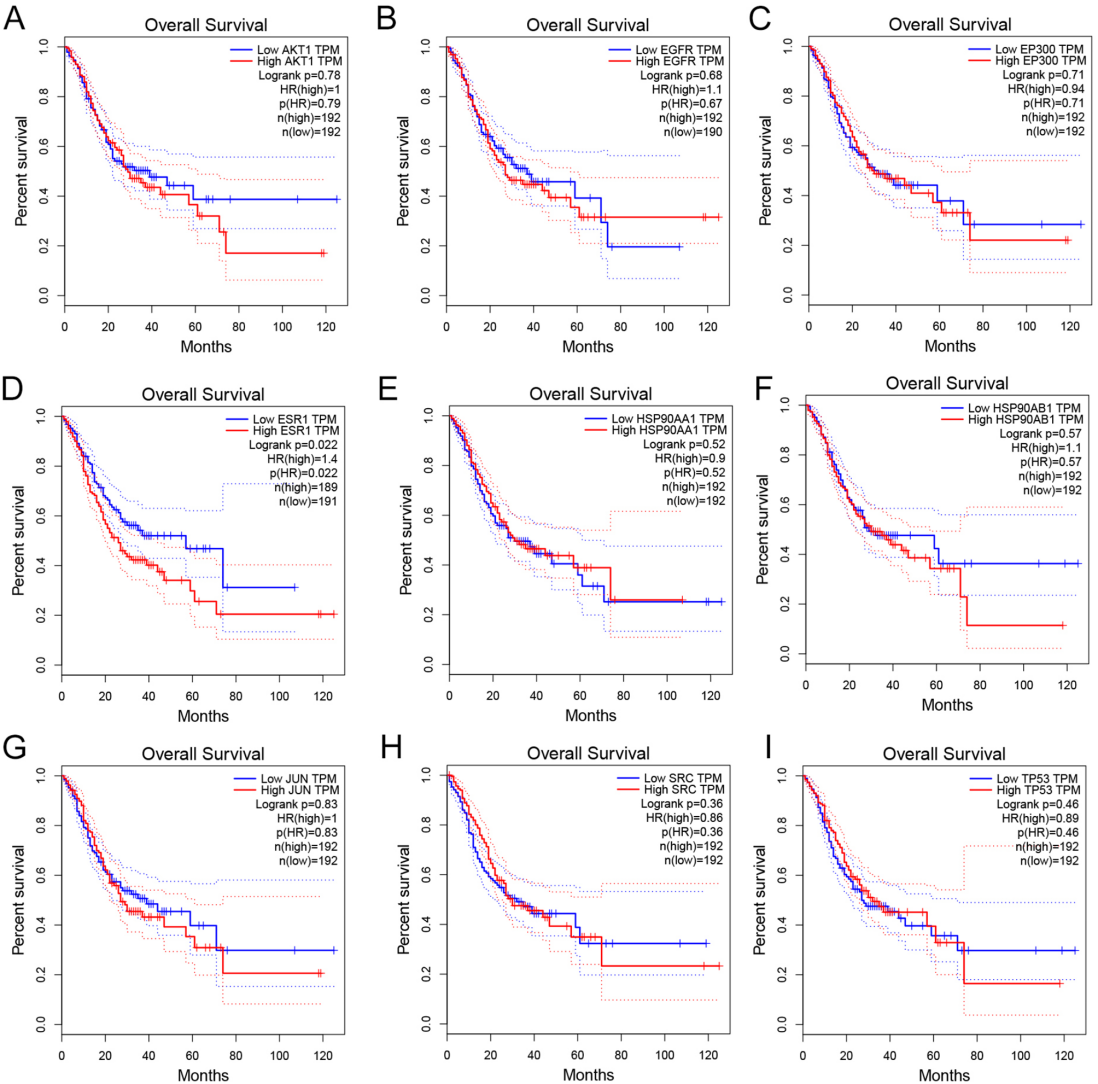
Relationship between core target expression and overall survival in the GEPIA database. Kaplan–Meier survival curves for AKT1 (**A**), EGFR (**B**), EP300 (**C**), ESR1 (**D**), HSP90AA1 (**E**), HSP90AB1 (**F**), JUN (**G**), SRC (**H**), and TP53 (**I**). The blue curve represents patients with low gene expression; the red curve represents those with high gene expression

Core target expression and clinical analysis through the GEPIA database showed that the expressions of *EP300* (Fig. 6C), *HSP90AA1* (Fig. 6E), *HSP90AB1* (Fig. 6F), *SRC* (Fig. 6H) and *TP53* (Fig. 6I) were upregulated in GC tissues compared to non-cancerous tissues (Fig. 6). Further investigation of gene expression across different tumor stages using GEPIA (Fig. 7) showed that ESR1 expression was significantly elevated in stages II, III, and IV (*P* = 0.0268, Fig. 7D). HSP90AA1 expression level decreased in stage II (*P* = 0.0359, Fig. 7E). JUN expression level increased in stages II and III but markedly reduced in stage IV(*P* = 0.0028, Fig. 7G). Additionally, TP53 expression was downregulated in stage IV(*P* = 0.0166, Fig. 7I). Kaplan-Meier survival curve demonstrated that high ESR1 expressions predicted the poor prognosis of GC patients (*P* = 0.022, Fig. 8).

### The results of molecular docking

**Fig. 9:**
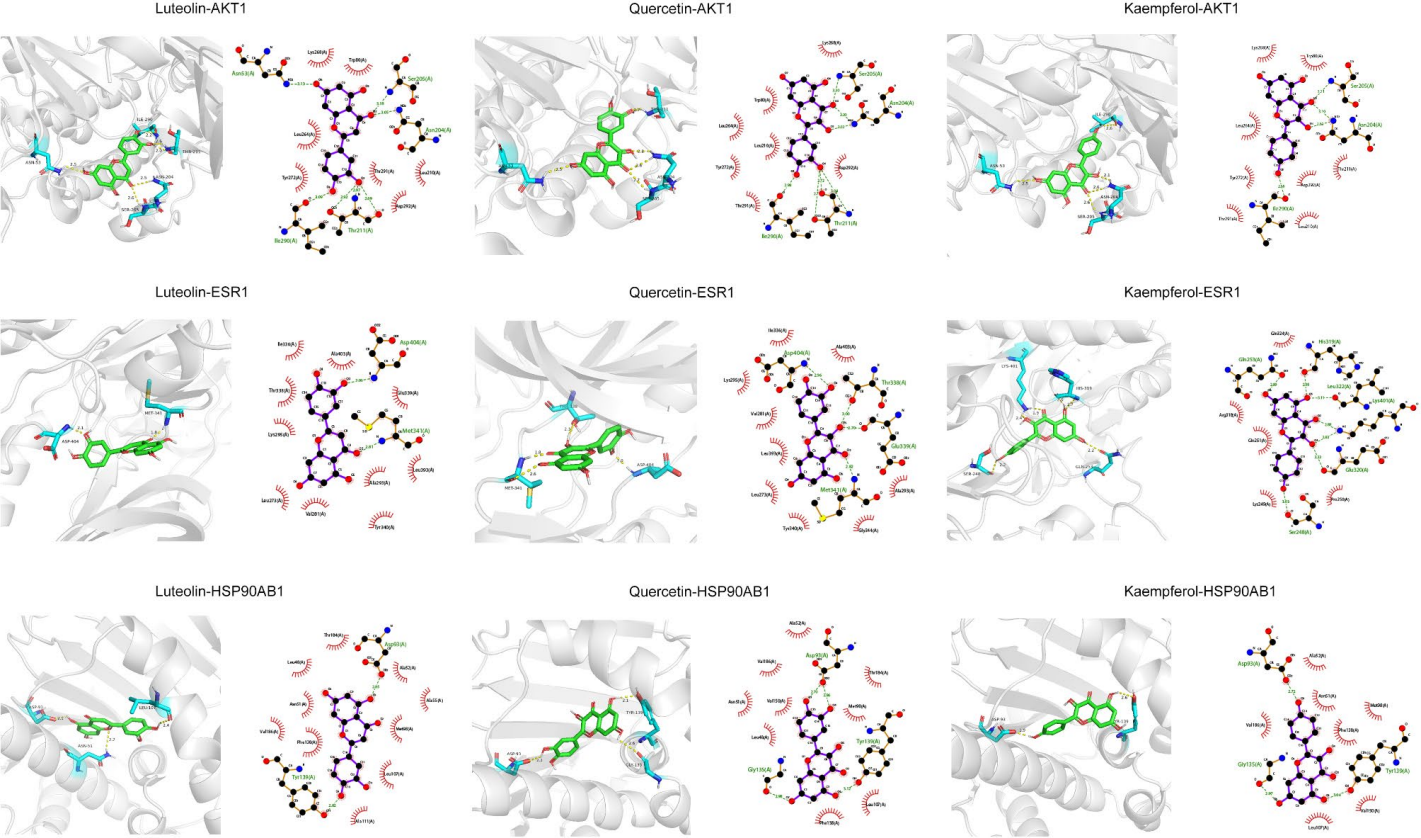
Molecular docking of luteolin, Quercetin and kaempferol combined with AKT1, ESR1 and HSP90AB1, respectively

Molecular docking analysis was performed to evaluate the binding interactions between active compounds in WQD and core target proteins. Quercetin, luteolin, and kaempferol exhibited strong binding affinities with all nine core proteins (binding energy <-5 kcal/mol, Table 3). Among them, luteolin showed strong interactions with AKT1, quercetin demonstrated a high affinity for ESR1, and kaempferol bound effectively to HSP90AB1 (Fig. 9). Luteolin formed seven hydrogen bonds with *Asn53*, *Ser205*, *Asn204*, *Ile290*, and *Thr211* on AKT1 and interacted with seven amino acid residues through hydrophobic interactions. When binding to ESR1, luteolin formed two hydrogen bonds with *Asp404* and *Met341* and engaged in hydrophobic interactions with seven additional residues. For HSP90AB1, luteolin established two hydrogen bonds with *Asp93* and *Tyr139*, and displayed hydrophobic interactions with ten residues. Quercetin formed four hydrogen bonds with *Asp404*, *Thr338*, *Glu339*, and *Met341* on ESR1, along with hydrophobic interactions involving nine residues. When binding to HSP90AB1, quercetin established four hydrogen bonds with *Asp93*, *Gly135*, and *Tyr139*, and exhibited hydrophobic interactions with nine residues. Kaempferol interacted with AKT1 through four hydrogen bonds with *Ser205*, *Asn204*, and *Ile290*, accompanied by hydrophobic interactions with eight residues. For ESR1, kaempferol formed seven hydrogen bonds with *Glu253*, *His319*, *Leu322*, *Lys104*, *Glu320*, and *Ser248*, along with hydrophobic interactions involving five residues. Binding to HSP90AB1 involved three hydrogen bonds with *Asp93*, *Gly135*, and *Tyr139* and hydrophobic interactions with seven residues.

**Table 3:** Docking results of hub targets and active ingredient

		Docking affinity score (kcal/mol)		
Target	PBD ID	Luteolin	Quercetin	Kaempferol
AKT1	7NH5	-10.0	-10.4	-9.8
EGFR	1M14	-7.7	-7.9	-7.6
EP300	5LPM	-7.9	-7.2	-8.0
ESR1	1A52	-9.0	-9.0	-7.7
HSP90AA1	1BYQ	-7.4	-7.0	-6.8
HSP90AB1	3NMQ	-9.0	-9.1	-8.8
JUN	1JUN	-5.5	-5.4	-5.3
SRC	1A07	-8.3	-8.3	-7.4
TP53	1A1E	-5.9	-5.8	-6.0

### WQD can inhibit the proliferation and migration of GC cells

**Fig. 10:**
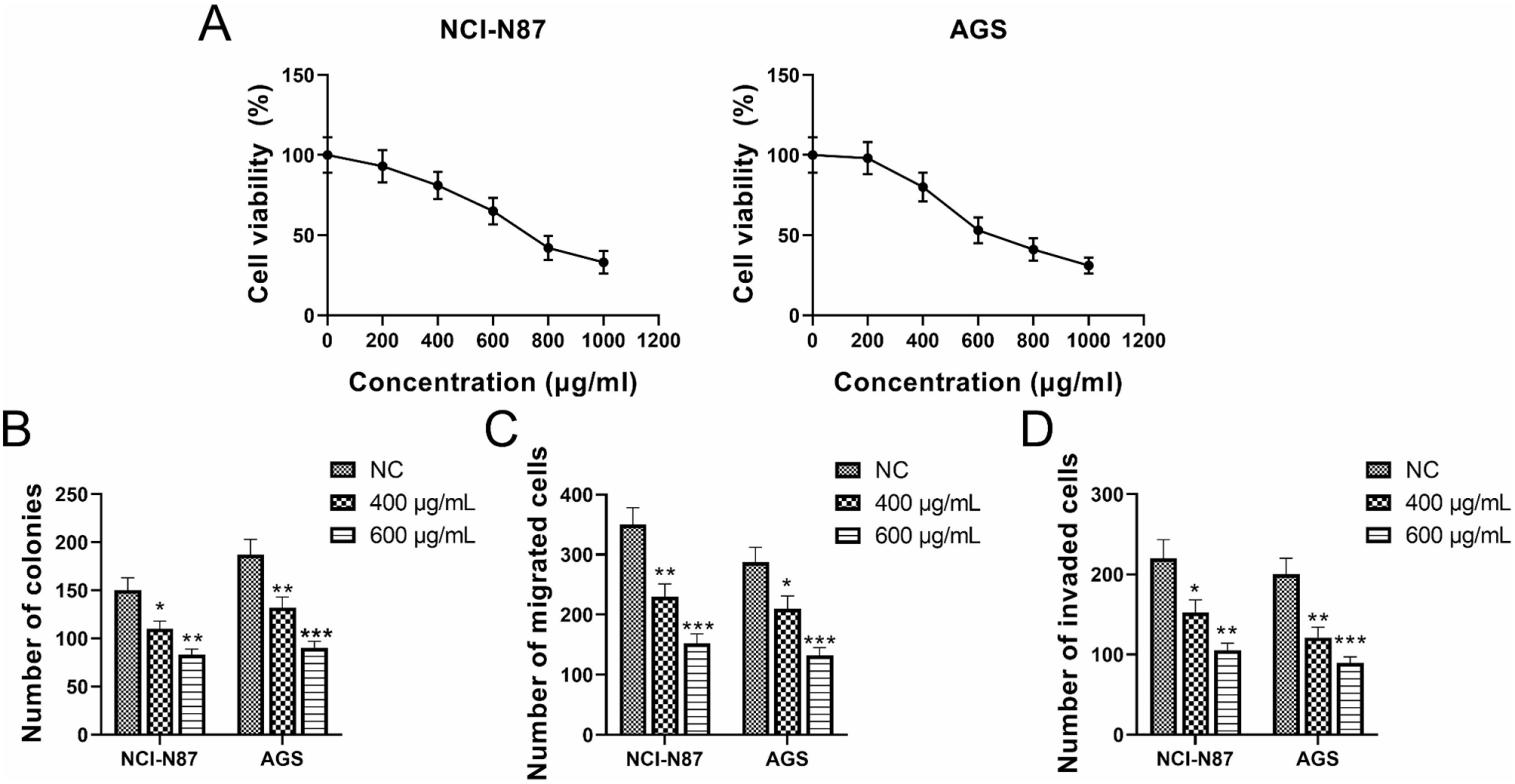
Inhibitory effects of WQD extract on GC cell proliferation, migration, and invasion. (**A**) CCK-8 assay was used to detect the viability of GC cells treated with WQD. (**B**) Colony formation assay was used to detect the proliferation of GC cells after WQD treatment. (**C**) Transwell assay was used to detect the cell migration ability of GC cells treated with WQD. (**D**) Transwell assay was used to detect the cell invasion ability of GC cells treated with WQD. *P < 0.05, **P < 0.01, and ***P < 0.001

The inhibitory effects of WQD on GC cells were evaluated to validate predictions from network pharmacological analyses. Treatment with WQD extract significantly reduced the viability of NCI-N87 and AGS cells in a dose-dependent manner, with progressive declines observed at concentrations ranging from 0 to 1000 µg/mL (Fig. 10A). The median inhibitory concentration (IC₅₀) values were 735.1 µg/mL for NCI-N87 cells and 680.9 µg/mL for AGS cells. Based on these results, concentrations of 400 µg/mL and 600 µg/mL were selected for subsequent functional assays. Colony formation assays revealed a marked reduction in the number of colonies following WQD treatment compared to the untreated control group (Fig. 10B). Transwell assays further demonstrated significant inhibition of cell migration and invasion (Fig. 10C-D). These findings suggest that WQD suppresses GC cell proliferation, migration, and invasion in vitro.

## Discussion

WQD is an empirical traditional Chinese formula developed through clinical practice in China for the treatment of chronic atrophic gastritis. It has been shown to effectively alleviate clinical symptoms, partially reverse gastric mucosal gland atrophy, and mitigate intestinal metaplasia [15]. Previous studies have reported that WQD regulates gastric mucosal blood flow disorders via the HIF-1α signaling pathway, thereby inhibiting the progression of chronic atrophic gastritis and its precancerous lesions [13]. Additionally, WQD suppresses AGS cell growth and induces cell cycle arrest at the G2/M phase [7]. However, the precise pharmacological mechanisms underlying its therapeutic effects against GC remain unclear.

Topological analysis of the drug-ingredient-target-disease network identified quercetin, luteolin, and kaempferol as the key active compounds in WQD against GC. Quercetin, a flavonol found in various vegetables and medicinal plants, has been shown to interact with multiple enzymes, including protein kinase C and cyclin-dependent kinases. It modulates the PI3K/Akt [16], JNK/p38 MAPK [17] and NF-κB signaling pathways [18], exerting anticancer, anti-inflammatory, and antioxidant effects [19]. Luteolin, a natural flavonoid with pro-apoptotic properties, has been recognized as a potential anti-tumor agent in various malignancies, including breast, lung, and GC [20–22]. It regulates key pathways such as AKT/mTOR, cytochrome c/caspase, and SGK1-FOXO3a-BNIP3, influencing tumor cell proliferation, metastasis, apoptosis, and autophagy [22–24]. Kaempferol, another widely distributed flavonoid, possesses anticancer properties, including anti-GC activity [25, 26]. Previous studies have shown that kaempferol induces autophagic cell death by inhibiting HDAC/G9a-mediated epigenetic modifications and activating the IRE1-JNK-CHOP pathway [27]. These findings suggest that quercetin, luteolin, and kaempferol are the principal bioactive components of WQD in GC treatment.

PPI network analysis identified *TP53*,* SRC*,* AKT1*,* HSP90AA1*,* ESR1*,* EP300*,* JUN*,* EGFR*, and *HSP90AB1* as key targets in WQD’s therapeutic effects against GC. TP53, a well-established tumor suppressor, regulates the cell cycle and apoptosis. Mutations in TP53 can impair its tumor-suppressive function and confer oncogenic properties, promoting malignant transformation, uncontrolled proliferation, and resistance to chemotherapeutic agents [28, 29]. SRC, a non-receptor tyrosine kinase, is significantly upregulated in GC tissues and contributes to multiple oncogenic processes, including cell proliferation, angiogenesis, apoptosis evasion, migration, and invasion [30]. AKT1, a serine/threonine-specific protein kinase, is involved in regulating glucose metabolism, cell growth, survival, and motility. Its aberrant activation has been implicated in GC tumorigenesis and progression [31]. HSP90AA1 and HSP90AB1, both subtypes of molecular chaperone HSP90, were significantly overexpressed in GC. Elevated expression of *HSP90AA1* was associated with shorter overall survival in patients, consistent with previous studies [32]. ESR1, encoding the estrogen receptor, plays a key role in GC pathogenesis through histone modification [33]. GEPIA database analysis showed that ESR1 expression was significantly elevated in advanced-stage GC and was negatively correlated with overall patient survival. EP300, a histone acetyltransferase, regulates transcription, cell cycle progression, proliferation, and differentiation through chromatin remodeling [34]. Notably, EP300 has also been implicated in conferring resistance to Apatinib in GC cells [35]. JUN, a component of the AP-1 transcription complex, is involved in tumor cell growth regulation [36]. Elevated expression of JUN has been associated with tumorigenesis and poor prognosis in various malignancies [37–39]. Data from the present study showed that JUN was significantly overexpressed in mid-stage GC, suggesting its potential involvement in disease progression. EGFR, a key upstream regulator of the MAPK/ERK signaling cascade, promotes tumor cell migration and invasion [40]. Numerous targeted cancer therapies have been developed against EGFR [41]. Molecular docking analysis demonstrated strong binding affinities of quercetin, luteolin, and kaempferol to these core targets, suggesting that WQD exerts antitumor effects through a multi-component, multi-target mechanism.

GO enrichment analysis indicated that WQD may exert therapeutic effects against GC by regulating key biological processes, including protein phosphorylation, negative regulation of apoptosis, and response to xenobiotic stimuli. KEGG pathway analysis further revealed that WQD primarily targets the AGE-RAGE, PI3K-Akt, and HIF-1 signaling pathways in the treatment of GC. The AGE-RAGE signaling pathway is known to activate downstream cascades such as NF-κB, MAPK, and JAK/STAT, thereby contributing to the inflammatory microenvironment and promoting GC cell proliferation and metastasis [42–44]. The PI3K/Akt pathway, one of the most frequently dysregulated pathways in human cancers, plays a crucial role in promoting GC cell proliferation, autophagy, and epithelial-mesenchymal transition (EMT), thus facilitating tumor progression [45–47]. The HIF-1 signaling pathway regulates genes involved in glycolytic metabolism, enhances the Warburg effect, and promotes GC cell migration and invasion under hypoxic conditions [48, 49]. These findings suggest that the active compounds in WQD exert anti-GC effects primarily by modulating these key signaling pathways, thereby inhibiting tumor growth and metastasis.

## Conclusion

This study investigated the molecular mechanisms of WQD in the treatment of GC by integrating network pharmacology, molecular docking, and in vitro experiments, thereby providing a theoretical foundation for its potential clinical application. However, certain limitations remain. The proposed mechanisms were primarily based on predictive analyses, and further experimental validation is necessary to confirm the specific interactions between the active compounds and their corresponding target proteins.

## Electronic supplementary material

Below is the link to the electronic supplementary material.

Supplementary Material 1

## Data Availability

The data used to support the findings of this study are available from the corresponding author upon request.
