# Supplementary material for: The mechanism of Weiqi decoction treating gastric cancer: a work based on network pharmacology and experimental verification
Source: Hereditas. 2025 Apr 21;162:67. doi: 10.1186/s41065-025-00434-3 (PMC12012975; doi:10.1186/s41065-025-00434-3)
Supplement: Supplementary file 1 — Supplementary Material 1 [file 41065_2025_434_MOESM1_ESM.pdf]

Supplementary Table 1 the primer sequence in RT-qPCR.

Gene		Sequence (5’-3’)
PI3K	Forward	AGACGAGGTGAGGAAGGAAGAGTG
	Reverse	GTCCTTGCCGTGGATGGTAAGC
AKT1	Forward	GGTTAGCCACTCTATCGCCATGAC
	Reverse	CCACAAGCCATTCTCCACTCCAC
RAGE	Forward	AAACATCACAGCCCGGATTG
	Reverse	TCCGGCCTGTGTTCAGTTTCT
HIF-1α	Forward	CCATTAGAAAGCAGTTCCGC
	Reverse	TGGGTAGGAGATGGAGATGC
VEGFA	Forward	AAGGAGGAGGGCAGAATCAT
	Reverse	ATCTGCATGGTGATGTTGGA
COX-2	Forward	CTGAATGGGGTGATGAGCAG
	Reverse	ATTCCTACCACCAGCAACCC
β-actin	Forward	CAGATGTGGATCAGCAAGCAGGA
	Reverse	CGCAACTAAGTCATAGTCCGCCTA

Supplementary Table 2 Information of active ingredients of WQD

Herbs	Molecule ID	Molecule name	MW	AlogP	Hdon	Hacc	OB (%)	Caco-2	BBB	DL	RBN
Danggui, Zhiqiao, Pugongying	MOL000358	beta-sitosterol	414.79	8.08	1	1	36.91	1.32	0.99	0.75	6

Danggui, Dangshen	MOL000449	Stigmasterol	412.77	7.64	1	1	43.83	1.44	1.00	0.76	5
Huangqi	MOL000211	Mairin	456.78	6.52	2	3	55.38	0.73	0.22	0.78	2
Huangqi	MOL000239	Jaranol	314.31	2.09	2	6	50.83	0.61	-0.22	0.29	3
Huangqi, ezhu	MOL000296	hederagenin	414.79	8.08	1	1	36.91	1.32	0.96	0.75	6
Huangqi	MOL000033	(3S,8S,9S,10R,13R,14S,17R)-10,13-dimethyl-17-[(2R,5S)-5-propan-2-yloctan-2-yl]-2,3,4,7,8,9,11,12,14,15,16,17-dodecahydro-1H-cyclopenta[a]phenanthren-3-ol	428.82	8.54	1	1	36.23	1.45	1.09	0.78	7
Huangqi	MOL000354	isorhamnetin	316.28	1.76	4	7	49.60	0.31	-0.54	0.31	2
Huangqi	MOL000371	3,9-di-O-methylnissolin	314.36	2.89	0	5	53.74	1.18	0.63	0.48	3
Huangqi	MOL000378	7-O-methylisomucronulatol	316.38	3.38	1	5	74.69	1.08	0.84	0.30	4
Huangqi	MOL000379	9,10-dimethoxypterocarpan-3-O-β-D-glucoside	462.49	0.74	4	10	36.74	-0.63	-1.50	0.92	5
Huangqi	MOL000380	(6aR,11aR)-9,10-dimethoxy-6a,11a-dihydro-6H-benzofurano[3,2-c]chromen-3-ol	300.33	2.64	1	5	64.26	0.93	0.55	0.42	2
Huangqi	MOL000387	Bifendate	418.38	2.56	0	10	31.10	0.15	-0.06	0.67	7
Huangqi	MOL000392	formononetin	268.28	2.58	1	4	69.67	0.78	0.02	0.21	2
Huangqi	MOL000398	isoflavanone	316.33	2.42	2	6	109.99	0.53	0.17	0.30	3
Huangqi	MOL000417	Calycosin	284.28	2.32	2	5	47.75	0.52	-0.43	0.24	2

Huangqi	MOL000422	kaempferol	286.25	1.77	4	6	41.88	0.26	-0.55	0.24	1
Huangqi	MOL000433	FA	441.45	0.01	7	13	68.96	-1.50	-2.59	0.71	9
Huangqi	MOL000438	(3R)-3-(2-hydroxy-3,4-dimethoxyphenyl)chroman-7-ol	302.35	3.13	2	5	67.67	0.96	0.34	0.26	7
Huangqi	MOL000442	1,7-Dihydroxy-3,9-dimethoxy pterocarpene	314.31	3.11	2	6	39.05	0.89	-0.04	0.48	2
Huangqi, Pugongying	MOL000098	quercetin	302.25	1.50	5	7	46.43	0.05	-0.77	0.28	1
Dangshen	MOL001006	poriferasta-7,22E-dien-3beta-ol	412.77	7.64	1	1	42.98	1.45	1.11	0.76	5
Dangshen	MOL002140	Perlolyrine	264.30	3.20	2	3	65.95	0.88	0.15	0.27	2
Dangshen	MOL002879	Diop	390.62	7.44	0	4	43.59	0.79	0.26	0.39	16
Dangshen	MOL003036	ZINC03978781	412.77	7.64	1	1	43.83	1.32	0.96	0.76	5
Dangshen	MOL003896	7-Methoxy-2-methyl isoflavone	266.31	3.36	0	3	42.56	1.16	0.56	0.20	2
Dangshen	MOL004355	Spinasterol	412.77	7.64	1	1	42.98	1.44	1.04	0.76	5
Dangshen	MOL005321	Frutinone A	264.24	2.70	0	4	65.90	0.89	0.46	0.34	0
Dangshen	MOL000006	luteolin	286.25	2.07	4	6	36.16	0.19	-0.84	0.25	1
Dangshen, Pugongying	MOL006554	Taraxerol	426.80	7.30	1	1	38.40	1.37	1.18	0.77	0
Dangshen	MOL006774	stigmast-7-enol	414.79	8.08	1	1	37.42	1.39	1.04	0.75	6
Dangshen	MOL007059	3-beta-Hydroxymethyllenetanshiquinone	294.32	3.16	1	4	32.16	0.38	-0.48	0.41	0
Dangshen	MOL007514	methyl icosa-11,14-dienoate	322.59	7.55	0	2	39.67	1.47	1.10	0.23	17
Dangshen	MOL008391	5alpha-Stigmastan-3,6-dione	428.77	6.66	0	2	33.12	0.90	0.47	0.79	6

Dangshen	MOL008397	Daturilin	436.64	4.34	0	4	50.37	0.61	0.06	0.77	1
Dangshen	MOL008400	glycitein	284.28	2.32	2	5	50.48	0.56	-0.29	0.24	2
Dangshen	MOL008407	(8S,9S,10R,13R,14S,17R)-17-[(E,2R,5S)-5-ethyl-6-methylhept-3-en-2-yl]-10,13-dimethyl-1,2,4,7,8,9,11,12,14,15,16,17-dodecahydrocyclopenta[a]phenanthren-3-one	410.75	7.31	0	1	45.40	1.49	1.26	0.76	5
Dangshen	MOL008411	11-Hydroxyrankinidine	356.46	1.04	2	6	40.00	0.29	-0.19	0.66	1
ezhu	MOL000906	wenjine	282.37	2.06	1.00	5	47.93	0.30	0.30	0.27	0
ezhu	MOL000940	bisdemethoxycurcumin	308.35	3.26	2.00	4	77.38	0.49	-0.08	0.26	6
Zhiqiao	MOL013381	Marmin	332.43	3.11	2	5	38.23	0.14	-0.46	0.31	7
Zhiqiao	MOL002341	Hesperetin	302.30	2.28	3	6	70.31	0.37	-0.25	0.27	2
Zhiqiao	MOL004328	naringenin	272.27	2.30	3	5	59.29	0.28	-0.37	0.21	1
Zhiqiao	MOL005828	nobiletin	402.43	3.04	0	8	61.67	1.05	-0.08	0.52	7
Pugongying	MOL004492	Chrysanthemaxanthin	584.96	8.24	2	3	38.72	0.51	-0.98	0.58	9
Pugongying	MOL002680	Flavoxanthin	584.96	8.24	2	3	60.41	0.97	-0.9	0.56	9
